# BREATHLESSNESS AND BIOSOCIALITY: AN ETHNOGRAPHY OF LIVING WITH RESPIRATORY DISEASE IN LATER LIFE

**DOI:** 10.1093/geroni/igad104.1889

**Published:** 2023-12-21

**Authors:** Fredrik Nyman

**Affiliations:** Mid Sweden University, Jönköping, Jonkopings Lan, Sweden

## Abstract

This paper is based on multi-sited ethnographic fieldwork conducted predominantly amongst support groups for older people with respiratory disease in the North of England, and sheds light on how communities are formed around chronic breathlessness. By utilising (and reconceptualising) the postmodern framework of biosociality, this paper explores how people living with respiratory disease negotiate and incorporate different kinds of health-related knowledge in their everyday lives—explicitly in support group settings outside of the clinic. People are living longer than ever before according to the World Health Organization, and chronic conditions are now the chief causes of death globally and have surfaced as major causes of disability and functional dependency. More specifically, in the United Kingdom 115,000 people die each year of chronic respiratory disease, which makes it one of the three biggest killer disease areas in the country. These mortality figures have remained stagnant for the past decades. What is more, in the era of neoliberalism respiratory care is individualised. Public health responses now emphasise the responsibility of individuals over collective or institutional responsibility, which is predominantly enforced through self-care and by training (or activating) patients in taking their medications and monitoring their pulmonary performance. By attending to public health responses in neoliberal times where respiratory healthcare regimens are habitually individualised, this paper contributes to understandings of biomedical subjectivities. Explicitly, it examines how support groups—as biosocial gatherings—can be understood as technologies for bridging dialogues between subjective and collective bodily experiences of health, illness, and wellbeing.

